# Supplementary material for: Cognition disconnected: the influence of domain-general cognition (g) in lesion-deficit mapping
Source: Cereb Cortex. 2026 Aug 20;36(8):bhag120. doi: 10.1093/cercor/bhag120 (PMC13490751; doi:10.1093/cercor/bhag120)
Supplement: Supplementary file 1 [file web_material_bhag120.docx]

Supplementary Material for

Cognition Disconnected: The Influence of Domain-General Cognition (g) in Lesion-Deficit Mapping

Lara Sozer^1^, Joseph Griffis^2^, Kathleen Langbehn^3^, Joel Bruss^2^, Emily Olafson^4^, Kenneth Manzel^3^, Maurizio Corbetta^5,6^, Daniel Tranel^3,7^, Aaron D. Boes^1,2,3^, & Mark Bowren^1,3,8,9^*

**Author affiliations:**

^1^Department of Psychiatry, Carver College of Medicine, University of Iowa, Iowa City, Iowa 52242, United States of America.

^2^Department of Pediatrics, Carver College of Medicine, University of Iowa, Iowa City, Iowa 52242, United States of America.

^3^Department of Neurology, Carver College of Medicine, University of Iowa, Iowa City, Iowa 52242, United States of America.

^4^Clinical Imaging Group, Genentech, South San Francisco, CA 94080, United States of America.

^5^Department of Neuroscience, Venetian Institute of Molecular Medicine, Padova, PD 32122, Italy.

^6^Padova Neuroscience Center, University of Padua, Padova, PD 32122, Italy.

^7^Department of Psychological and Brain Sciences, University of Iowa, Iowa City, Iowa 52242, United States of America.

^8^Department of Neurosurgery, Carver College of Medicine, University of Iowa, Iowa City, Iowa 52242, United States of America.

^9^Iowa Neuroscience Institute, Iowa City, Iowa 52242, United States of America.

Correspondence to: Mark Bowren, Jr.

T221 GH, 200 Hawkins Drive, Iowa City, IA 52242

mark-bowren@uiowa.edu

PLSR model visualization methods

For each PLSR model, the sign of the cognitive factor (i.e., the behavioral input into the PLSR model) was flipped so that positive model weights were associated with lower factor scores. Visualizations in figures and downstream analyses were based on the positive normalized regression weights to allow for a lesion-deficit interpretation of the results. We used a False Discovery Rate (FDR) correction (corrected alpha = 0.05) to threshold the model weights for visualization whenever possible. If no model weights survived the FDR correction, we used an alternative threshold to facilitate visual comparison of the correlated factors- and bifactor-based PLSR models: if one map in a given cognitive domain and predictor modality (e.g., correlated factors version of LBM of verbal learning/memory) had model weights that did not survive FDR correction, but its counterpart from the other psychometric model (e.g., the bifactor version of LBM of Gl) did, we applied the model weight threshold from the latter to the former so that the relative importance of the model features could be more easily compared across the maps. If neither map in a given cognitive domain and predictor modality had model weights that survived FDR correction, we applied a threshold of 0.2 to both sets of normalized voxel weights.

Additional information on structural equation modeling procedures

We built the baseline model (Model 1) based upon previously published work in a smaller subsample of the Iowa Registry^1^ in which observed variables were assigned to load on to g and the following factors based on the CHC taxonomy: crystalized intelligence (Gc), visuospatial ability (Gv), learning/memory (Gl), and processing speed (Gs). Tests that were not included in prior work were fit into the baseline model using prior work and the CHC model^2^ as a guide where possible: Boston Naming Test loaded on Gc, Benton Facial Recognition Test and the Matrix Reasoning subtest from the Wechsler Adult Intelligence Scales (WAIS) loaded on Gv, and the Symbol Search subtest from the WAIS loaded on Gs. Because multiple new tests of language were included (Boston Naming Test from the Boston Diagnostic Aphasia Examination (BDAE), and Token Test, Sentence Repetition and Controlled Oral Word Association from the Multilingual Aphasia Examination (MAE)), we also modeled a language factor (LANG) using these tests in Model 1. We then tested for the added value of modeling the covariance between WAIS subtests that belong to the same composite score domain (Model 2), the covariance among the domain-specific factors (i.e., removing orthogonality constraints on domain-specific factors; Model 3), and the following hypothesized cross-loadings that have not been previously tested (Model 4): Benton Visual Retention Test loading on Gl given the memory aspect of the task demands, COWA loading on Gs given the speed demands of the test, and WRAT loading on LANG due to its reading demands. We then examined the local fits (i.e., the statistical significance of parameter estimates) of the best-fitting model, and re-fit that model after removing parameters that did not have an associated *p*-value of less than 0.05. This final model was used to perform lesion-deficit mapping and calculate model-based reliability estimates for each latent factor using the omega-hierarchical coefficient as calculated by the compRelSEM function in R’s lavaan package.

For presenting the modeling results in a figure, we used abbreviations for the domain-specific factors derived from the CHC model of cognitive abilities (Schneider and McGrew 2018a); otherwise, for clarity in the main text, we refer to each factor by its full name. The CHC model has been shown to fit neuropsychological test data well (Jewsbury et al. 2017; Schneider and McGrew 2018b).

Additional structural equation modeling results

Model 1 (the baseline model) provided a somewhat poor fit to the data (Robust CFI = 0.888, Robust TLI = 0.862, RMSEA = 0.079, SRMR = 0.085). The addition of the covariances between WAIS subtests belonging to the same composite score domain (Model 2) resulted in a non-positive definite variance-covariance matrix, and thus was rejected in favor of Model 1. The addition of non-orthogonal covariances among domain-specific factors (Model 3; Robust CFI = 0.931, Robust TLI = 0.911, Robust RMSEA = 0.063, SRMR = 0.054) fit the data significantly better than Model 1 (Χ^2^ Difference = 142.99, *p* < 0.001), and thus Model 3 was retained over Model 1. The addition of hypothesized cross-loadings (Model 4; Robust CFI = 0.947, Robust TLI = 0.930, Robust RMSEA = 0.056, SRMR = 0.048) fit the data better than Model 3, and was retained over Model 3 (Χ^2^ Difference = 82.72, *p* < 0.001). We re-fit Model 4 after removing the following non-significant parameters: covariances between Gc and Gs, Gc and LANG, Gv and Gl, and Gs and LANG. The final, re-fit model provided a good to excellent fit to the data (Robust CFI = 0.948, Robust TLI = 0.933, Robust RMSEA = 0.055, SRMR = 0.054) and did not statistically significantly differ in model fit to the data compared to the original Model 4 (Χ^2^ Difference = 4.97, *p* < 0.547).

Lastly, we reported here the model-based reliability estimates (omega hierarchical) for each domain-specific factor because these are often of interest for psychometric purposes. In the final bifactor model, omega-hierarchical coefficients for the latent variables were: g (0.594), crystalized intelligence (0.335), verbal learning/memory (0.567), processing speed (0.450), visuospatial ability (0.587), and language (0.250). For the final correlated factors model, these coefficients were: crystalized intelligence (0.618), verbal learning/memory (0.610), processing speed (0.712), visuospatial ability (0.745), and language (0.524).

Methods for LBM, sLNM, and fLNM of individual tests

To test whether g’s influence on LBM and LNM was limited to the level of factor scores, we created mass-univariate lesion-deficit maps in which individual tests were the outcome variables rather than the cognitive factor scores in the primary analyses. We compared mass-univariate models with and without regressing out g. We did so for one test from each of the non-g domains, using the test with the highest factor loading in each domain in the Iowa correlated factors model: Wechsler Adult Intelligence Scale (WAIS) Block Design for visuospatial ability, Rey Auditory-Verbal Learning Test (RAVLT) Trial 5 for verbal learning/memory, WAIS Symbol Search for processing speed, and Token Test for language. We also performed these analyses for the Controlled Oral Word Association (COWA) test from the Multilingual Aphasia Examination to serve as an expressive language analogue to the Token Test maps, which were used to evaluate whether controlling for g was effectively just controlling for deficits in expressive or receptive language. Because each test was used to model the original g scores in the primary structural equation modeling results, we recalculated the g factor for the purposes of mapping each of the tests described above. Specifically, we re-estimated g after removing the g loading of the test being mapped. For example, to perform lesion-deficit mapping of Block Design while controlling for g, we first calculated g after removing the loading of Block Design onto g in the bifactor model. This g estimate was thus created without involving the Block Design test data, thus keeping the confound variable separate from the dependent variable; this was the g estimate used as a confound for the LBM, sLNM, and fLNM analyses in which we mapped Block Design while controlling for g. Each model was fit using data from participants who completed the test in question (i.e., we omitted missing data), resulting in different sample sizes for the Block Design (n = 467), Token Test (n = 372), RAVLT Trial 5 (n = 459), Symbol Search (n = 281), and COWA (n = 468) models.

Additional Case Examples from an Independent Cohort of Patients with Focal Brain Lesions

In order to provide additional case examples about the potential utility of the bifactor maps relative to the correlated factors maps, we acquired case examples from a separate cohort of patients with focal brain lesions. Our hypothesis was that patients who had a brain lesion involving a bifactor (BF) map but not its correlated factors (CF) counterpart would present with more selective domain-specific deficits. For example, if we were to use a lesion-behavior map of visuospatial ability without controlling for g (CF map), the map would include areas critical for visuospatial ability along with areas for g. Because of this, the CF map could not be used to identify those individuals with *selective* deficits to the exclusion of individuals with broad deficits. Conversely, we hypothesized that a patient with a lesion intersecting only a CF map (but not it’s BF counterpart) would show more generalized deficits (broadly low scores) that could account for low domain-specific scores due to the fact that the CF map included domain-general regions that could be attributed to g. To test these ideas, we acquired data from an independent dataset of patients with focal brain lesions from Washington University in St. Louis.^3^ This dataset has been described in detail elsewhere, and included patients with first-time, unilateral ischemic stroke. We present neuropsychological test data from this cohort (collected during the first two weeks after stroke onset) for tests that had normatively adjusted T scores or percentile scores: the Hopkins Verbal Learning Test-Revised (HVLT-R) Immediate Recall Total Score, Delayed Recall, and Discrimination Index, the Brief Visuospatial Memory Test-Revised (BVMT-R) Immediate Recall Total and Delayed Recall, the Spatial Span subtest (both forward and backward spans) from the Wechsler Memory Scales Third Edition (WMS-III), Animal Naming (i.e., category fluency for animals), and the following subtests from the Boston Diagnostic Aphasia Examination (BDAE): Word Comprehension, Commands, Complex Ideational Material, Boston Naming Test (short form), Oral Reading of Sentences, and Comprehension of Oral Reading of Sentences.

We overlapped each Washington University patient’s lesion mask from the Washington University cohort with the LBMs of the domain-specific factors generated from the Iowa cohort data (i.e., the primary dataset for the study). We searched for patients who had a lesion mask that intersected the BF version of an LBM and not the CF version of the LBM, and conversely, patients with a lesion mask that intersected the CF version of an LBM but not its BF counterpart. Once patients were identified on this basis, we examined their neuropsychological test scores described above, which were converted to a Z score metric for comparability across tests. Supplementary Figure 8 depicts the case examples from this dataset where the patient’s lesion intersected either a BF map only or a CF map only. Panel A shows a case in which a patient’s lesion intersected the BF but not CF lesion-behavior map of visuospatial ability, and Panel B presents data from a patient whose lesion intersected the CF map for learning/memory (but not the BF version of this map).

Because the Washington University cohort comprises patients with unilateral focal stroke patients, the lesion distribution was such that group-level analyses comparing BF-only and CF-only intersection groups would have been limited by a low number of cases in one or both groups. That being said, for the domain of learning/memory, we note that 16 patients had a lesion mask that intersected the Iowa CF version of the LBM and not BF version of the LBM, and conversely, 3 patients had a lesion mask that intersected the BF version of LBM of learning/memory from Iowa cohort but not the CF version of the LBM. The CF-only intersection group demonstrated a substantially lower average Z score across neuropsychological tests (mean Z = -0.699) relative to that of the patients who only intersected the bifactor version of the map (mean Z = 0.097). This finding is consistent with the notion that the CF version of the learning/memory map included anatomy that led to broadly lower scores across neuropsychological tests, not just in tests of memory.

Partitioning out g Is Not Equivalent to Regressing Out Expressive or Receptive Language Deficits

We performed analyses to evaluate whether controlling for g was, in effect, controlling for expressive or receptive language. First, we mapped both the Token Test and COWA with and without using g as a covariate. The g factor was calculated without the test in question as an indicator of the latent g variable in order to keep the covariate separate from variable of interest. For the Token Test, canonical receptive language anatomy (i.e., left posterior superior temporal region / Wernicke’s area) was present in the lesion-behavior map both with and without controlling for g (Supplementary Figure 13A). Similarly, canonical expressive language anatomy (i.e., the left inferior frontal region / Broca’s area) was present in LBM of COWA both with and without controlling for g (Supplementary Figure 13B). Taken together, these LBM results suggest that partitioning out g-associated variance is not simply equivalent to controlling for expressive or receptive language deficits.

Lastly, we calculated the within-domain change in spatial correlation among the LBMs for each domain. Consistent with prior left-lateralized findings for g and closely associated cognitive abilities like cognitive control (Barbey et al. 2012; Bowren et al. 2020; Jiang et al. 2023), these results demonstrated greater change in intra-domain spatial correlation among language tests (mean change in cross-test spatial correlation = 0.244) relative to those of processing speed (0.078), learning/memory (0.035), and visuospatial ability (-0.093).

Supplementary Figure 1 Lesion Overlap Map. The spatial distributions of the lesion masks is depicted. Voxel values indicate the number of lesion masks present in that location.

Supplementary Figure 2 sLNM of g Controlling for Lesion Chronicity at Behavioral Testing. The sLNM of g was performed again with lesion chronicity at behavioral testing (determined as the time of administration of the first set of WAIS subtests) as the covariate in the mass-univariate model.

**Supplementary Figure 3 PLSR-Based Lesion-Deficit Mapping of g**. To complement the mass-univariate analyses, PLSR was used to generate LBM, sLNLM, and fLNM results for the g factor from the bifactor psychometric model shown in Figure 2.

**Supplementary Figure 4 PLSR-Based Lesion-Deficit Mapping of Visuospatial Ability**. To complement the mass-univariate analyses, PLSR was used to generate LBM, sLNLM, and fLNM results for the visuospatial ability factors from the psychometric models.

**Supplementary Figure 5 PLSR-Based Lesion-Deficit Mapping of Processing Speed**. To complement the mass-univariate analyses, PLSR was used to generate LBM, sLNLM, and fLNM results for the processing speed factors from the psychometric models.

**Supplementary Figure 6 PLSR-Based Lesion-Deficit Mapping of Verbal Learning/Memory**. To complement the mass-univariate analyses, PLSR was used to generate LBM, sLNLM, and fLNM results for the verbal learning/memory factors from the psychometric models.

**Supplementary Figure 7 PLSR-Based Lesion-Deficit Mapping of Language**. To complement the mass-univariate analyses, PLSR was used to generate LBM, sLNLM, and fLNM results for the language factors from the psychometric models.

**Supplementary Figure 8 Additional Case Examples.** (A) A patient from the WU cohort had a lesion that intersected the bifactor (BF) but not correlated factors (CF) lesion-behavior map of visuospatial ability. This patient’s test scores were notably lower for the Spatial Span Forward task (a test of visuospatial working memory span and attention) relative to their average level of performance across all tests (gray dashed line). (B) A second patient from the WU cohort had a lesion that intersected the CF map for learning/memory, but not the BF version of this map. This patient’s mean test score across domains was notably low (gray dashed line) – nearly 2 standard deviations below the normative mean. The low memory test scores for this patient may be best interpreted as reflecting generally low cognitive performances (or low g) that is driving low scores on memory tests – not a circumscribed memory deficit per se. This is notable because this patient’s lesion did not involve medial temporal structures, which were identified in both maps. This nuance would be lost if one were to focus on the CF lesion-behavior map of learning/memory alone, as this map did not exclude the g-linked regions.

Supplementary Figure 9 LBM and LNM of WAIS Block Design. Lesion-Behavior Mapping, and Structural and Functional Lesion Network Mapping results are presented for the Wechsler Adult Intelligence Scale (WAIS) Block Design subtest, both with and without controlling for a version of domain-general cognition (g) derived without the use of the Block Design data (see supplemental methods). Differences between the two sets of results are highlighted.

Supplementary Figure 10 LBM and LNM of RAVLT Trial 5. Lesion-Behavior Mapping, and Structural and Functional Lesion Network Mapping results are presented for the Rey Auditory-Verbal Learning (RAVLT) Trial 5 subtest, both with and without controlling for domain-general cognition (g), which was derived without the use of the RAVLT Trial 5 data.

Supplementary Figure 11 LBM and LNM of WAIS Symbol Search. Lesion-Behavior Mapping, and Structural Lesion Network Mapping results are presented for the Wechsler Adult Intelligence Scale (WAIS) Symbol Search subtest, both with and without controlling for domain-general cognition (g), which was derived without the use of the Symbol Search data. Functional Lesion Network Mapping did not yield any statistically significant results.

Supplementary Figure 12 LBM and LNM of Token Test. Structural and Functional Lesion Network Mapping results are presented for the Token Test with and without controlling for domain-general cognition (g), which was derived without the use of the Token Test data.

Supplementary Figure 13 LBM of Token Test and COWA with and without Controlling for g. LBM of Token Test (panel A) showed that canonical receptive language anatomy (posterior superior temporal gyrus / Wernicke’s area) was present in the result both before and after controlling for g. Similarly, LBM of COWA (panel B) implicated canonical expressive language anatomy (left inferior frontal gyrus / Broca’s area) both before and after controlling for g. Together, these results suggest that controlling for g does not equate to controlling for expressive or receptive language deficits.

Supplementary Table 1 Neuropsychological and Cognitive Tests.

WAIS Similarities*

WAIS Information*

WAIS Block Design**

WAIS Matrix Reasoning**

WAIS Digit Span*

WAIS Arithmetic*

WAIS Digit-Symbol Coding**

WAIS Symbol Search**

WRAT Word Reading*

Benton Judgment of Line Orientation**

Benton Facial Recognition Test**

Benton Visual Retention Test – Total Correct**

Rey-Osterrieth Complex Figure Test – Copy**

Rey-Osterrieth Complex Figure Test – Delayed Recall**

Rey AVLT Trial 5*

Rey AVLT Delayed Recall*

Rey AVLT Recognition Hits*

BDAE Boston Naming Test*

MAE Token Test*

MAE Sentence Repetition*

MAE Controlled Oral Word Association*

Trail-Making Test Part A**

Trail-Making Test Part B**

Note: *Principally verbally mediated test; **Principally visually mediated test.

**Supplementary Table 2** Spatial Correlations of Mass-Univariate Lesion-Deficit Mapping Results Controlling for Non-g Covariates.

|  | Bifactor Model-No Covariate | Bifactor Model-Controlling for Lesion Volume | Correlated Factors Model-No Covariate | Correlated Factors Model-Controlling for Age | Correlated Factors Model-Controlling for Lesion Volume |
| --- | --- | --- | --- | --- | --- |
| LBM | -0.018 | -0.056 | 0.297 | 0.213 | 0.299 |
| sLNM | 0.055 | -0.072 | 0.551 | 0.388 | 0.551 |
| fLNM | 0.159 | 0.273 | 0.353 | 0.385 | 0.536 |
| Spatial correlations are based on unthresholded model weights; LBM = Lesion-Behavior Mapping; sLNM = Structural Lesion Network Mapping; fLNM = Functional Lesion Network Mapping; SD = Standard Deviation. | | | | | |

**Supplementary Table 3** Spatial Correlations of Mass-Univariate Lesion-Deficit Mapping Results from Each Bifactor Map and Its Correlated Factors Counterpart.

|  | LBM | sLNM | fLNM | Mean (SD) Across Predictor Modalities |
| --- | --- | --- | --- | --- |
| Verbal Learning/Memory | 0.918 | 0.909 | 0.822 | 0.883 (0.043) |
| Processing Speed | 0.588 | 0.692 | 0.845 | 0.708 (0.106) |
| Visuospatial Ability | 0.873 | 0.791 | 0.819 | 0.828 (0.034) |
| Language | 0.949 | 0.926 | 0.754 | 0.876 (0.087) |
| Mean (SD) Across Factors | 0.832 (0.143) | 0.830 (0.095) | 0.810 (0.034) | 0.824 (0.070) |
| Spatial correlations are based on unthresholded model weights; LBM = Lesion-Behavior Mapping; sLNM = Structural Lesion Network Mapping; fLNM = Functional Lesion Network Mapping; SD = Standard Deviation. | | | | |

.

**Supplementary Table 4** Anatomy Associated with Top Cluster Peaks from LBM of Cognitive Factors.

| Cluster Peak # | 1 | 2 | 3 | 4 | 5 | 6 | 7 |
| --- | --- | --- | --- | --- | --- | --- | --- |
| g | L TPOJ1 (x=-48, y=-45, z=12; r=0.383) | L A4 (x=-57, y=-27, z=9; r=0.376) | CPT O L (x=-33, y=-48, z=12; r=0.374) | L A5 (x=-66, y=-30, z=3; r=0.374) | L A4 (x=-69, y=-30, z=9; r=0.373) | DRTT L / RST L / TR S L (x=-24, y=12, z=30; r=0.233) | L 6v / SLF2 L (x=-57, y=-6, z=42; r=0.225) |
| Gl-CF | CC  (x=-42, y=-48, z=0; r=0.294) | L STSvp / PAT L / AF L (x=-51, y=-45, z=-3; r=0.268) | IFOF L / ILF L (x=-42, y=-36, z=-6; r=0.267) | CC (x=-45, y=-30, z=-9; r=0.266) | L TE1p / PAT L / AF L (x=-60, y=-51, z=-3; r=0.260) | C PH L (x=-21, y=-33, z=-12; r=0.258) | L Ig / CS S L (x=-33, y=-9, z=18; r=0.246) |
| Gl-BF | L PHA2 / C PH L (x=-30, y=-36, z=-12; r=0.328) | L PHA1 (x=-24, y=-39, z=-15; r=0.327) | C PH L / C PHP L (x=-21, y=-33, z=-15; r=0.324) | L V3 (x=-21, y=-69, z=-12; r=0.304) | L V4 (x=-21, y=-75, z=-6; r=0.300) | F L (x=-33, y=-24, z=-9; r=0.212) | IFOF L (x=-30, y=9, z=-6; r=0.207) |
| Gs-CF | CC (x=-24, y=-51, z=21; r=0.276) | CC (x=-21, y=-51, z=27; r=0.268) | VOF L / IFOF L / ILF L (x=-24, y=-87, z=9; r=0.260) | CC (x=-27, y=-57, z=27; r=0.258) | MdLF L (x=-24, y=-57, z=39; r=0.257) | CC (x=-21, y=-81, z=18; r=0.249) | CC (x=-27, y=-69, z=15; r=0.243) |
| Gs-BF | R MST (x=48, y=-69, z=6; r=0.247) | CC (x=30, y=-51, z=12; r=0.240) | TR P R / IFOF R / ILF R (x=36, y=-57, z=6; r=0.237) | CC (x=27, y=-69, z=24; r=0.230) | CPT O L (x=-21, y=-81, z=12; r=0.228) | TR S R (x=21, y=-24, z=21; r=0.226) | CC (x=-18, y=-87, z=6; r=0.226) |
| Gv-CF | IFOF R / ILF R (x=36, y=-57, z=9; r=0.261) | CC (x=30, y=-60, z=9; r=0.251) | R Putamen (x=33, y=-12, z=0; r=0.250) | R PGs / SLF2 R (x=42, y=-66, z=42; r=0.250) | CC (x=30, y=-48, z=15; r=0.247) | CC (x=27, y=-45, z=21; r=0.247) | R PGs (x=45, y=-75, z=36; r=0.245) |
| Gv-BF | R Putamen (x=33, y=-12, z=0; r=0.295) | ILF R / IFOF R (x=36, y=-57, z=9; r=0.283) | EMC R (x=36, y=3, z=-9; r=0.276) | ML R (x=24, y=-27, z=24; r=0.276) | CC (x=27, y=-45, z=21; r=0.271) | ILF R (x=36, y=-63, z=9; r=0.268) | CC (x=30, y=-48, z=15; r=0.266) |
| LANG-CF | L A4 (x=-69, y=-30, z=9; r=0.522) | L PFop (x=-63, y=-24, z=15; r=0.514) | L A4 (x=-66, y=-18, z=12; r=0.488) | L STV (x=-66, y=-39, z=15; r=0.486) | L TE1a (x=-66, y=-18, z=-12; r=0.302) | CC (x=-18, y=-57, z=33; r=0.289) | L V2 (x=-24, y=-102, z=0; r=0.289) |
| LANG-BF | L A4 (x=-69, y=-27, z=9; r=0.453) | L A4 (x=-66, y=-18, z=12; r=0.43) | CC (x=-42, y=-33, z=0; r=0.403) | L STV (x=-66, y=-45, z=15; r=0.400) | L 45 (x=-39, y=27, z=3; r=0.343) | L AVI (x=-30, y=27, z=6; r=0.336) | RST L / IFOF L (x=-42, y=27, z=9; r=0.333) |
| CF = Correlated Factors Mode; BF = Bifactor Model; Gv = Visuospatial Ability; Gl = Verbal Learning/Memory; Gs = Processing Speed; LANG = Language; Coordinates are in MNI152 1mm space; Anatomical descriptors were derived from the Human Connectome Project (HCP) Multimodal Parcellation Atlas Version 1 and the Harvard-Oxford Subcortical Atlas for results in cortical and sub-cortical regions, and the HCP 1065 group-averaged white matter atlas for results in white matter; w = Partial Least Squares Regression Weight Value; L = Left Hemisphere; R = Right Hemisphere. | | | | | | | |

**Supplementary Table 5** Cortical Parcel Pairs Associated with Top Model Weights from sLNM of Cognitive Factors.

| Weight # | 1 | 2 | 3 | 4 | 5 | 6 | 7 |
| --- | --- | --- | --- | --- | --- | --- | --- |
| G | L SomMot 4 and L Limbic TempPole 2 (r=0.397) | L Cont Par 1 and L Default Temp 4 (r=0.395) | L SomMot 6 and L DosAttn Post 2 (r=0.393) | L Cont Par 1 and L Cont Temp 1 (r=0.392) | L SomMot 4 and L Cont Temp 1 (r=0.391) | L SomMot 6 and L Cont Temp 1 (r=0.391) | L SomMot 4 and L Default Temp 4 (r=0.390) |
| Gl-CF | L Vis 9 and L Default PFC 5 (r=0.324) | L Vis 14 and L Default PFC 5 (r=0.322) | L Vis 2 and L Default PFC 3 (r=0.322) | L Vis 7 and LH SalVentAttn FrOperIns2 (r=0.321) | L Vis 13 and L Default PFC 5 (r=0.320) | L Vis 14 and L Default PFC 3 (r=0.319) | L Vis 11 and L Default PFC 5 (r=0.318) |
| Gl-BF | L Vis 2 and L Limbic TempPole 2 (r=0.327) | L Vis 7 and L Limbic TempPole 2 (r=0.326) | L Vis 4 and L Limbic TempPole 2 (r=0.323) | L Default pCunPCC1 and L Default PHC 1 (r=0.319) | L Vis 6 and L Default PHC 1 (r=0.316) | L Vis 10 and L Limbic TempPole 2 (r=0.314) | L Vis 1 and L Limbic TempPole 2 (r=0.311) |
| Gs-CF | L Vis 7 and R Vis 14 (r=0.317) | L Default Temp 4 and R Vis 12 (r=0.317) | L Default Temp 3 and R Vis 13 (r=0.313) | L Vis 7 and R Vis 12 (r=0.310) | L Default Temp 3 and R Vis 3 (r=0.309) | L Default Temp 4 and R Vis 13 (r=0.307) | L Vis 13 and R Vis 14 (r=0.304) |
| Gs-BF | L Vis 7 and R Vis 12 (r=0.328) | L Vis 7 and R Vis 14 (r=0.326) | L Vis 7 and R Vis 4 (r=0.319) | L Vis 7 and R Vis 9 (r=0.319) | L Vis 13 and R Vis 6 (r=0.317) | L Vis 13 and R Vis 12 (r=0.315) | L Vis 14 and R Vis 9 (r=0.311) |
| Gv-CF | L DorsAttnPost 8 and R Vis 5 (r=0.287) | L DorsAttnPost 9 and R Default Temp 5 (r=0.280) | L DorsAttnPost 8 and R Vis 15 (r=0.276) | L DorsAttnPost 7 and R DorsAttnPost 1 (r=0.276) | L DorsAttn Post 3 and R Vis 5 (r=0.274) | L DorsAttn Post 7 and R Vis 154 (r=0.271) | L DorsAttn Post 3 and R DorsAttn Post 2 (r=0.271) |
| Gv-BF | R Vis 12 and R Limbic OFC 2 (r=0.334) | R Vis 12 and R Cont PFCv 1 (r=0.330) | R Vis 14 and R Cont PFCv 1 (r=0.328) | R Vis 12 and R Default PFCv 1 (r=0.325) | R Vis 12 and R SalVentAttn FrOperIns4 (r=0.322) | R Vis 6 and R Default PFCv 1 (r=0.321) | R Vis 15 and R Default PFCv 1 (r=0.320) |
| LANG-CF | L SomMot4 and L Limbic TempPole 2 (r=0.513) | L Vis 13 and L SalVentAttn FrOperIns2 (r=0.490) | L SomMot 2 and L SomMot 4 (r=0.487) | L Vis 13 and L Default PFC 5 (r=0.486) | L Vis 14 and L Default PFC 5 (r=0.486) | L SomMot 4 and L Default Temp 4 (r=0.482) | L Vis 11 and L Default PFC 5 (r=0.482) |
| LANG-BF | L SalVentAttn ParOper 2 and left thalamusproper (r=0.385) | L Default Temp 5 and left thalamus proper (r=0.382) | L SalVentAttn ParOper 1 and left thalamus proper (r=0.377) | L SomMot 5 and left thalamus proper (r=0.372) | L SomMot 4 and L Limbic TempPole2 (r=0.368) | L Vis 9 and L Default PFC 5 (r=0.367) | L Vis 7 and L Default PFC 3 (r=0.365) |
| Anatomical descriptors were derived from existing atlases spanning cortical, subcortical, and brainstem regions as described in the methods section of the primary text; CF = Correlated Factors Model; BF = Bifactor Model; Gv = Visuospatial Ability; Gl = Verbal Learning/Memory; Gs = Processing Speed; LANG = Language; r = Pearson Correlation Coefficient Value; L = Left Hemisphere; R = Right Hemisphere. | | | | | | | |

**Supplementary Table 6** Anatomy Associated with Top Cluster Peaks from fLNM of Cognitive Factors.

| Cluster Peak # | 1 | 2 | 3 | 4 | 5 | 6 | 7 |
| --- | --- | --- | --- | --- | --- | --- | --- |
| g | TR S L / SLF3 L / AF L (x=-42, y=-42, z=30; r=0.334) | CC (x=-54, y=-54, z=6; r=0.322) | L TPOJ2 (x=-45, y=-54, z=12; r=0.316) | AF L / FAT L (x=-42, y=12, z=15; r=0.271) | RST L / IFOF L (x=-42, y=27, z=9; r=0.270) | CB R (x=18, y=-63, z=-27; r=0.249) | V (x=21, y=-72, z=-45; r=0.248) |
| Gl-CF | L PuM / OR L (x=-12, y=-33, z=0; r=0.235) | CB R (x=21, y=-75, z=-21; r=0.224) | CC (x=-9, y=21, z=15; r=0.218) | CB R (x=39, y=-60, z=-24; r=0.218) | IFOF L (x=-42, y=-42, z=-9; r=0.209) | CS A L (x=-24, y=33, z=6; r=0.207) | V (x=15, y=-75, z=-42; r=0.200) |
| Gl-BF | L PuM / OR L (x=-12, y=-33, z=0; r=0.284) | CC (x=-9, y=24, z=12; r=0.236) | CB R (x=27, y=-78, z=-21; r=0.231) | CB R (x=9, y=-81, z=-18; r=0.226) | L V1 (x=31, y=22, z=26; r=0.221) | CB R (x=45, y=-60, z=-24; r=0.217) | L PHA3 (x=-30, y=-39, z=-18; r=0.213) |
| Gs-CF | F L (x=-21, y=-36, z=6; r=0.259) | L PuM (x=-15, y=-30, z=3; r=0.254) | CC (x=-18, y=-66, z=36; r=0.246) | OR L (x=-30, y=-30, z=0; r=0.245) | L IP1 (x=-27, y=-69, z=36; r=0.241) | L VVC (x=-33, y=-33, z=-30; r=0.241) | F R (x=24, y=-36, z=6; r=0.240) |
| Gs-BF | F L (x=-21, y=-36, z=6; r=0.370) | R PuM (x=21, y=-33, z=6; r=0.362) | CC (x=0, y=9, z=18; r=0.287) | CC (x=-18, y=-12, z=30; r=0.217) | CPT P R (x=9, y=-30, z=-27; r=0.195) | - | - |
| Gv-CF | C PHP R  (x=18, y=-60, z=39; r=0.193) | R TE2a (x=57, y=-42, z=-27; r=0.192) | CC (x=3, y=6, z=21; r=0.186) | CC (x=-18, y=-12, z=30; r=0.182) | CPT F R / CS S R / TR S R / FAT R (x=27, y=6, z=24; r=0.182) | CC (x=9, y=-6, z=27; r=0.181) | - |
| Gv-BF | R PuM / OR R (x=18, y=-33, z=6; r=0.276) | V (x=-9, y=-75, z=-39; r=0.246) | CC (x=6, y=-6, z=24; r=0.245) | C PHP R (x=21, y=-60, z=33; r=0.242) | C PHP R (x=18, y=-63, z=39; r=0.240) | CC (x=9, y=-75, z=45; r=0.234) | ICP L (x=-9, y=-66, z=-30; r=0.231) |
| LANG-CF | AF L (x=-42, y=-45, z=9; r=0.354) | IFOF L (x=-45, y=30, z=6; r=0.314) | IFOF L (x=-33, y=36, z=-3; r=0.303) | CB R (x=18, y=-69, z=-24; r=0.298) | MCP (x=-9, y=-18, z=-33; r=0.272) | L TE2p (x=-48, y=-51, z=-24; r=0.243) | L TE2p (x=-51, y=-39, z=-24; r=0.236) |
| LANG-BF | CC (x=-42, y=-42, z=6; r=0.254) | PAT L / AF L (x=-42, y=-48, z=15; 2=0.237) | CB R (x=18, y=-69, z=-24; r=0.219) | L TE1p (x=-48, y=-57, z=-21; r=0.214) | - | - | - |
| CF = Correlated Factors Mode; BF = Bifactor Model; Gv = Visuospatial Ability; Gl = Verbal Learning/Memory; Gs = Processing Speed; LANG = Language; Coordinates are in MNI152 1mm space; Anatomical descriptors were derived from the Human Connectome Project (HCP) Multimodal Parcellation Atlas Version 1 and the Harvard-Oxford Subcortical Atlas for results in cortical and sub-cortical regions, and the HCP 1065 group-averaged white matter atlas for results in white matter; r = Pearson Correlation Coefficient Value; L = Left Hemisphere; R = Right Hemisphere. | | | | | | | |

**Supplementary Table 7** PLSR-Based Modeling Results.

| Outcome Variable | Predictor Modality | Inferential Model Proportion Variance Explained |
| --- | --- | --- |
| g (residualized on log lesion volume) | LBM | 0.198^***^ |
|  | sLNM | 0.215^***^ |
|  | fLNM | 0.191^***^ |
| Verbal Learning/Memory – Correlated Factors (residualized on log lesion volume) | LBM | 0.130^*^ |
|  | sLNM | 0.204^***^ |
|  | fLNM | 0.124^***^ |
| Processing Speed – Correlated Factors (residualized on log lesion volume) | LBM | 0.199 |
|  | sLNM | 0.121^**^ |
|  | fLNM | 0.043^***^ |
| Visuospatial Ability – Correlated Factors (residualized on log lesion volume) | LBM | 0.094 |
|  | sLNM | 0.075^**^ |
|  | fLNM | 0.227^*^ |
| Language – Correlated Factors (residualized on log lesion volume) | LBM | 0.413^***^ |
|  | sLNM | 0.361^***^ |
|  | fLNM | 0.269^***^ |
| Verbal Learning/Memory – Bifactor (residualized on log lesion volume) | LBM | 0.106 |
|  | sLNM | 0.126^**^ |
|  | fLNM | 0.078^***^ |
| Processing Speed – Bifactor (residualized on log lesion volume) | LBM | 0.236^*^ |
|  | sLNM | 0.177^**^ |
|  | fLNM | 0.114^***^ |
| Visuospatial Ability– Bifactor (residualized on log lesion volume) | LBM | 0.396^***^ |
|  | sLNM | 0.182^***^ |
|  | fLNM | 0.167^***^ |
| Language – Bifactor (residualized on log lesion volume) | LBM | 0.257^***^ |
|  | sLNM | 0.245^***^ |
|  | fLNM | 0.184^***^ |

References

1.	Bowren M Jr, Adolphs R, Bruss J, et al. Multivariate lesion-behavior mapping of general cognitive ability and its psychometric constituents. *J Neurosci*. 2020;40(46):8924-8937.

2.	Schneider WJ, McGrew KS. The Cattell-Horn-Carroll Theory of Cognitive Abilities. In: Flanagan DP, McDonough EM, eds. 4th ed. Guilford Press; 2018:72-163.

3.	Corbetta M, Ramsey L, Callejas A, et al. Common behavioral clusters and subcortical anatomy in stroke. *Neuron*. 2015;85(5):927-941.
